# Tattoo-induced immunologic reaction to red inks

**DOI:** 10.5415/apallergy.0000000000000165

**Published:** 2024-11-05

**Authors:** João Vieira, João Marcelino, Sofia Farinha, Miguel Proença, Joana Guimarães, Rui Bajanca, Elza Tomaz

**Affiliations:** 1 Immunoallergology Department, Unidade Local de Saúde da Arrábida, Setúbal, Portugal; 2 Institute of Allergology, Charité – Universitätsmedizin Berlin, corporate member of Freie Universität Berlin and Humboldt-Universität zu Berlin, Berlin, Germany; 3 Fraunhofer Institute for Translational Medicine and Pharmacology ITMP, Allergology and Immunology, Berlin, Germany; 4 Immunoallergology Department, Unidade Local de Saúde de Lisboa Ocidental, Lisboa, Portugal; 5 Dermatology Department, Unidade Local de Saúde da Arrábida, Setúbal, Portugal

**Keywords:** Patch tests, red ink, tattoo reaction

## Abstract

A 43-year-old male presented with pruritic nodular lesions in the red dye area of his leg tattoo, which developed 4 weeks after its application. Patch tests were performed using a standard series, and the inks used by the tattooist were tested semi-open. Tests identified a sensitization to 2 inks containing an azo-organic dye (Pigment Red 170), diketopyrrolopyrrole (Pigment Red 254), and copper phthalocyanine (Pigment Blue 15). Histopathological findings suggested a pseudolymphoid reaction, likely driven by T-cell hypersensitivity to the red pigments. Although the utility of patch testing in the assessment of tattoo reactions is not consensual, it can be useful in identifying the offending inks, helping to guide future tattoo choices and prevent recurrences. Patch testing including the suspected ink should not be disregarded from the diagnostic workup.

## 1. Introduction

Tattoos are increasingly popular and have been associated with both cutaneous and systemic complications. Tattoo allergic reactions may present in a variety of clinical and histological patterns [1]. Even though black is the most often used color for tattoos, red ink is the most frequently linked to adverse reactions [2]. Historically, the red pigment was manufactured by using cinnabar (mercuric sulfide), a known sensitizer. In today’s red inks, this compound is seldom used in favor of red azo dyes and quinacridones, also associated with adverse reactions [1]. Assessment of tattoo-related reactions can be challenging, and the utility of patch testing is not consensual [3].

## 2. Case report

**Figure 1.**
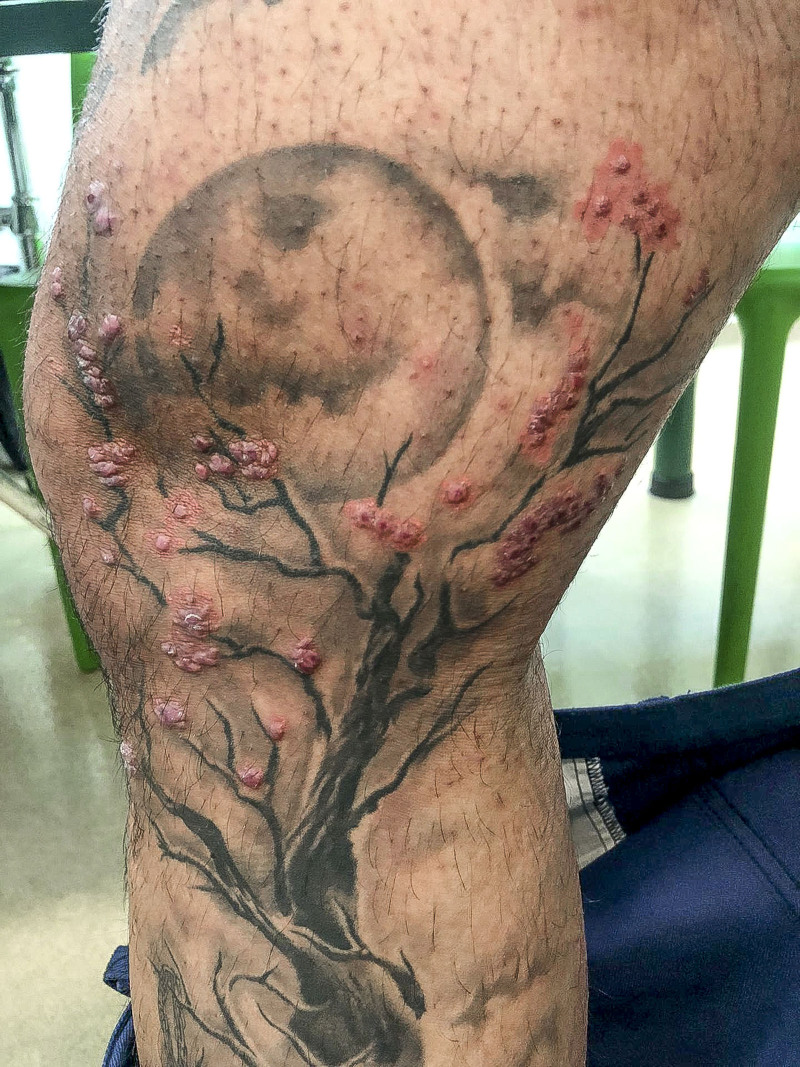
Picture of the patient’s limb showing nodular lesions where the red ink was applied.

We present a case of a 43-year-old male who, 4 weeks after getting a black and red tattoo on his leg, developed pruritic nodular lesions in the regions of red dye application (Fig. 1). The patient had previous tattoos, some with red ink, but which remained unaffected. The patient was initially medicated with topical betamethasone valerate and fusidic acid with modest improvement.

Patch tests were performed and read according to the European Society of Contact Dermatitis recommendations: patches were placed on the upper back of the patient, followed by a 48-hour occlusion period, and readings on days 2 and 4. The inks used by the tattooist were tested semi-open, applied undiluted on the skin, allowed to dry completely, and covered with permeable tape. The patient was tested using the Portuguese Society of Allergy and Clinical Immunology standard series (Chemotechnique Diagnostics, Vellinge, Sweden). The tested inks were (1) Bright Red from Intenze Products, composed of Pigment Red (PR) 210 (CI 12477); (2) Dark Red from Intenze Products, a mixture of PR 254 (CI 56110) and Pigment Blue (PB) 15 (CI 74160); (3) Solid Ink, with nonspecified pigments; and (4) Red Wagon from Vintage Ink, composed of PR 170 (CI 12475). Additionally, water, glycerin, propanol, and hamamelis virgin extract were present in all of the inks.

**Figure 2.**
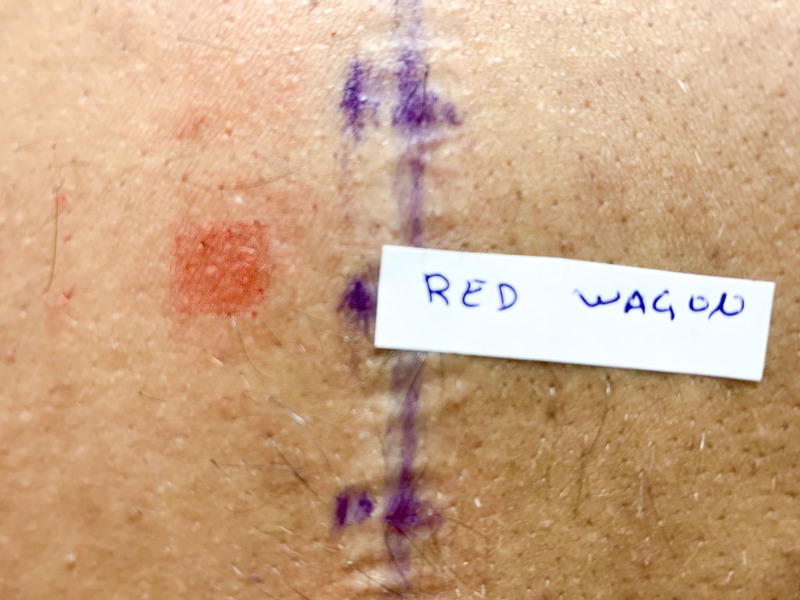
Patch test showing a positive result to the Red Wagon ink on day 4 (96 hours reading).

The tests were positive on day 4 for Dark Red ink (+) and Red Wagon ink (++) (Fig. 2). The patient refused to perform further patch tests with the metal and colors series.

**Figure 3.**
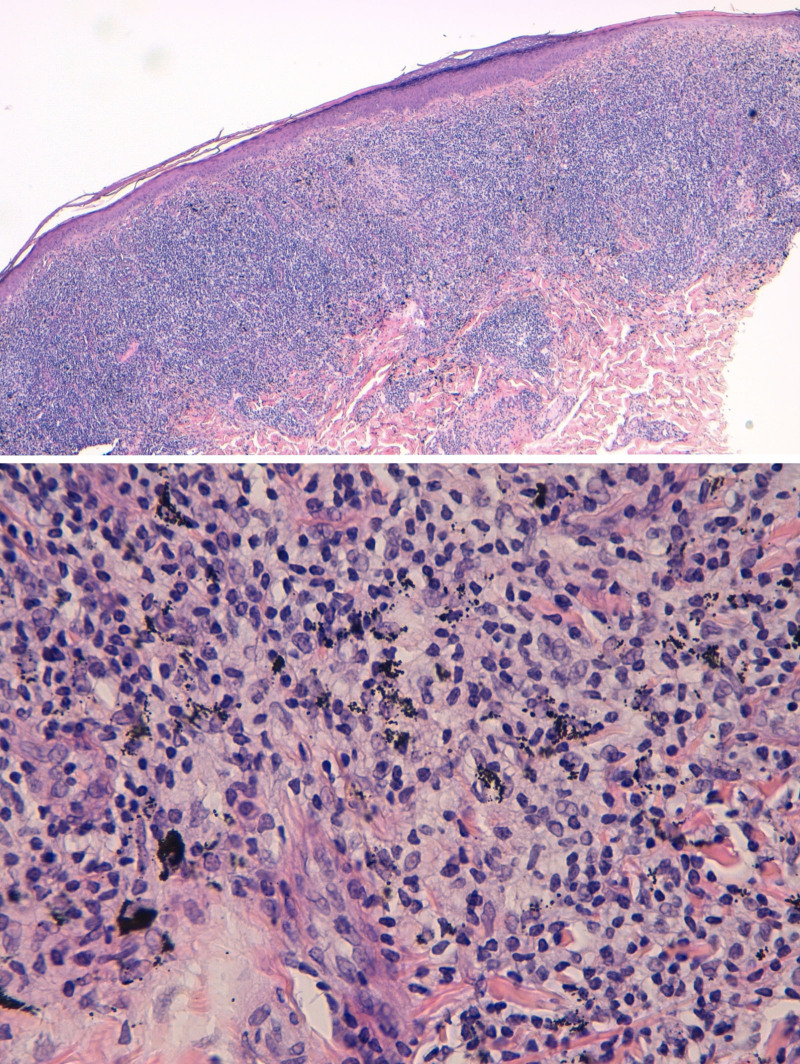
Histopathologic images of the lesion on the patient’s red ink tattoo after surgical removal, demonstrating a band-like lichenoid infiltrate, with granulomatous and pseudolymphoid changes.

Due to financial constraints, laser tattoo removal was not a possibility. The tattoo was surgically removed with success. Skin biopsy showed a band-like lichenoid infiltrate, with interface abnormalities, and granulomatous and pseudolymphoid changes. The infiltrate consisted mainly of T cells and some B cell aggregates (Fig. 3).

## 3. Discussion

Patch testing is the standard procedure for the diagnosis of type IV hypersensitivity reactions. However, there are inconsistent results regarding its utility in tattoo reactions [1–4]. Previous studies suggest that patch testing had limited usefulness in demonstrating allergic sensitization to specific inks/pigments, as many yielded negative results on patch tests. This may be due to the formation of new allergens from the pigment by slow haptenization in the skin [3].

In this report, we present a clinical case of a cutaneous reaction to red tattoo ink. The patient demonstrated sensitization to 2 inks containing an azo-organic dye (PR 170), diketopyrrolopyrrole (PR 254), and copper phthalocyanine (PB 15). It is important to note that tattoo inks are crude industrial products with varying levels of purity, and positive patch test results could be due to contaminants, such as metals in red inks, making it difficult to identify the precise chemical constituent responsible for the reaction [3]. Despite this challenge, patch testing played an important role in highlighting the inks involved in the reaction, offering guidance for future tattoo choices.

Histopathological examination revealed findings consistent with a pseudolymphoid reaction, likely mediated by T-cell hypersensitivity to the red pigments. Although the precise mechanisms remain unclear, it is thought that the chronic antigen stimulation caused by the red exogenous pigment causes a proliferation of lymphoid cells [5]. Given the complexity of these reactions, the broader term “tattoo-induced immunologic reaction” has been proposed to encompass the diverse clinical presentations [2].

While patch testing should be interpreted with caution and alongside a thorough histopathological evaluation, it may provide valuable insights for patients with tattoo-related skin reactions. It should not be disregarded from the diagnostic workup. We recommend testing the suspected ink.

The European Union implemented restrictions on tattoo ink composition in January 2022. However, there are no limitations on red pigment use. Efforts must be made to further identify and regulate potentially harmful components in tattoo inks.

## Conflicts of interest

The authors have no financial conflicts of interest.

## Author contributions

João Vieira: conceptualization, writing—original draft. João Marcelino: writing—review and editing. Sofia Farinha: resources. Miguel Proença and Joana Guimarães: investigation. Rui Bajanca: resources. Elza Tomaz: supervision, project administration.
